# Epigenetic Signature of Childhood Adversity in Older Adults

**DOI:** 10.1093/geroni/igab046.2507

**Published:** 2021-12-17

**Authors:** Eric Klopack, Eileen Crimmins

**Affiliations:** University of Southern California, Los Angeles, California, United States

## Abstract

Past research has shown that childhood adversity (CA) affects the health of older adults; however, the biological mechanisms underlying this association are unclear. Though past research has implicated DNA methylation (DNAm), studies utilizing representative data from older adults and reliable DNAm measures are needed to answer key questions about how stable DNAm changes associated with CA are in later life. Methylation risk scores (MRSs) are an emerging tool that can be used as biomarkers of exposure and as a dimension reduction approach for mediation analyses. This study clarifies the association between CA and later life health by generating MRSs for childhood adversity based on an epigenome wide association study conducted in an independent sample and validating that measure in a nationally representative sample of older adults living in the US from the Health and Retirement Study (HRS), including 2016 methylation data from the HRS Innovative Subsample of the Venous Blood Study. For these 4,018 respondents, DNAm was assessed in whole blood using the Illumina Infinium Human Methylation EPIC BeadChip microarray. Results indicate that retrospective report of childhood SES is significantly associated with an MRS for CA after controlling for demographic factors (viz., race and ethnicity, age, gender, smoking status, and BMI), suggesting that DNAm signal from CA persists across the life course into older adulthood. This study helps clarify the biological processes underlying the association between CA and adult health.

